# Single-dose dexamethasone increases perioperative glycemic variability in older patients with diabetes undergoing lung surgery: a continuous glucose monitoring-based randomized controlled trial

**DOI:** 10.3389/fmed.2026.1880028

**Published:** 2026-08-27

**Authors:** Xiaoyi Zhang, Cheng Huang, Xiang Wang, Jun Zhang, Xiaomei Ling, Jiahao Xu, Jue Ma, Fei Zhong, Haihua Shu

**Affiliations:** 1 Guangdong Cardiovascular Institute, Guangdong Provincial People’s Hospital, Guangdong Academy of Medical Sciences, Guangzhou, Guangdong, China; 2 Department of Anesthesiology, Guangdong Provincial People’s Hospital (Guangdong Academy of Medical Sciences), Southern Medical University, Guangzhou, Guangdong, China; 3 Global Health Research Center, Guangdong Provincial People’s Hospital (Guangdong Academy of Medical Sciences), Southern Medical University, Guangzhou, Guangdong, China

**Keywords:** continuous glucose monitoring, dexamethasone, elderly, glycemic variability, type 2 diabetes mellitus

## Abstract

**Objectives:**

To investigate whether a single intraoperative dose of dexamethasone alters perioperative glycemic dynamics in older patients with type 2 diabetes undergoing thoracoscopic lung resection. Glycemic dynamics were captured using continuous glucose monitoring (CGM).

**Methods:**

72 patients aged ≥ 60 years with type 2 diabetes who underwent elective thoracoscopic lung resection were randomly assigned to receive either 8 mg of dexamethasone or placebo (0.9% saline) intravenously during anesthesia induction. The primary outcome was the maximum change in intraoperative blood glucose from preoperative baseline. Key secondary outcomes included CGM-derived glycemic variability (GV) metrics, including standard deviation (SD), coefficient of variation (CV), blood glucose instability index (GLI) and time-in-range measures, assessed throughout the perioperative period. Multivariable linear regression was performed to assess the independent effect of dexamethasone on glycemic outcomes after adjusting for relevant covariates. The accuracy of CGM was assessed using mean absolute relative difference and Bland-Altman analysis.

**Results:**

The dexamethasone group exhibited greater median (IQR) maximum intraoperative glucose change: 3.6 (3.3–4.9) mmol/L vs. 3.1 (2.6–4.0) mmol/L; *P* = 0.009. Dexamethasone was also associated with higher intraoperative GV: SD (1.1 [1.0–1.4] mmol/L vs. 0.8 [0.7–1.0] mmol/L, *P* < 0.001), CV (21.9 [6.3] vs. 15.8 [6.1]; *P* < 0.001), and GLI (3.0 [1.7–4.1] mmol^2^/L^2^/h vs. 1.4 [0.9–2.4] mmol^2^/L^2^/h; *P* < 0.001). Multivariable analysis confirmed that dexamethasone independently predicted greater intraoperative glucose excursions and glycemic variability. Perioperative time-in-range metrics did not differ significantly between groups. CGM demonstrated acceptable accuracy in this surgical setting.

**Conclusion:**

A single 8 mg intraoperative dose of dexamethasone increases maximum intraoperative glucose change and amplifies GV in older patients with diabetes. These findings reveal a previously uncharacterized dimension of dexamethasone-induced glycemic instability in this vulnerable population and highlight the need for glycemic monitoring strategies that capture transient fluctuations potentially missed by conventional intermittent measurements.

**Clinical trial registration:**

www.chictr.org.cn, identifier [ChiCTR2300077167].

## Introduction

Dexamethasone is widely used perioperatively for its antiemetic (1), opioid-sparing, and anti-inflammatory effects (2). However, as a long-acting glucocorticoid steroid, it has the potential to induce hyperglycemia by stimulating hepatic gluconeogenesis and increasing insulin resistance (3). Accordingly, concerns have arisen regarding its safety for high-risk patients with diabetes.

Evidence on the glycemic impact of a single dose of dexamethasone remains conflicting, and dedicated data in older patients with type 2 diabetes mellitus (T2DM) are particularly scarce. Some small-scale studies have demonstrated that dexamethasone can significantly increase postoperative blood glucose levels (4–6), indicating the need for caution when prescribing it to in individuals with diabetes. While a meta-analysis indicated that a single prophylactic dose for postoperative nausea and vomiting (PONV) led to only a modest increase in peak postoperative glucose levels within 24 h among surgical patients with diabetes, supporting its safety in this population (7). Conversely, another clinical trial detected no significant changes in blood glucose concentrations (8). Critically, most prior studies have not focused specifically on older patients, who represent a growing proportion of the surgical population and in whom perioperative glycemic management is especially challenging due to higher comorbidity burdens and reduced physiological reserve.

Furthermore, conventional blood glucose monitoring provides only intermittent data and may miss transient glycemic fluctuations. Continuous glucose monitoring (CGM) offers a comprehensive, real-time glucose profile and has become a established tool for glycemic management in patients with diabetes (9). The high density of CGM data allows for a more precise quantification of glycemic variability (GV) and captures subtle patterns of dysglycemia that cannot be obtained with isolated readings. However, its application in the perioperative setting remains limited (10), partly due to concerns regarding its accuracy under surgical conditions. In this study, we therefore employed CGM as the primary glucose monitoring method, with its accuracy validated against standard measurements to ensure data reliability.

To the best of our knowledge, no prior study has specifically characterized the perioperative glycemic profiles of dexamethasone in older patients with diabetes. This randomized trial was designed to address this gap, using CGM to capture glycemic dynamics with high resolution.

## Materials and methods

### Study design

This prospective randomized controlled trial was approved by the Medical Research Ethics Committee of Guangdong Provincial People’s Hospital (Approval No. KY2023-622-02). The trial was registered with the Chinese Clinical Trial Registry (ChiCTR2300077167, www.chictr.org.cn) and was conducted at Guangdong Provincial People’s Hospital between November 2023 and May 2024. The study was conducted in accordance with the Declaration of Helsinki and reported following the Consolidated Standards of Reporting Trials (CONSORT) 2010 guidelines. Written informed consent was obtained from all participants.

### Patients

We enrolled patients aged 60 years or older with a clinical diagnosis of T2DM who were scheduled for elective video-assisted thoracoscopic lung resection under general anesthesia, with an expected surgical duration of 1.5–4 h. Exclusion criteria included: critical illness involving the heart, liver, kidneys or other vital organ dysfunction; serious neurological or psychiatric disorders (e.g., schizophrenia, epilepsy, Parkinson’s disease); American Society of Anesthesiologists (ASA) physical status ≥ IV; preoperative fasting blood glucose exceeding 10 mmol/L (11), recent diabetic ketoacidosis, hyperglycemia hyperosmolar state or severe hypoglycemia within 3 months prior to surgery; known allergy to hormonal agents or history of long-term hormone use; preoperative chemoradiotherapy for tumors; emergency surgery; planned postoperative transfer to the intensive care unit; current participation in another interventional clinical trial; unsuitability for CGM use; any other condition deemed unsuitable by the investigators.

### Randomization and blinding

Randomization codes were generated using SPSS (version 22.0; IBM, Armonk, NY, United States). Participants were randomized in a 1:1 ratio to receive either dexamethasone or a matched placebo and each participant was assigned a unique allocation number. A non-investigating anesthetist prepared the study drugs after opening a sealed opaque envelope containing the assigned allocation. The responsible anesthesiologist was informed of the allocated treatment due to the risk of dexamethasone-induced intraoperative hyperglycemia in older patients with diabetes. However, intraoperative insulin administration followed a standardized protocol and was guided by point-of-care capillary glucose measurements. CGM data were accessible only to the research team and all clinical decisions were independent of CGM readings. Surgeons, outcome assessors, and patients remained blinded to the allocation throughout the study, with unblinding permitted only when clinically imperative.

### CGM system and glucose monitoring

The GS1 CGM system (Shenzhen Sibionics Technology Co. Ltd.)-comprising a sensor, reader, and system software (12)-was utilized and factory-calibrated. Glucose measurements were recorded every 5 min for up to 14 days. Staff received training on the CGM device and the glucose data application. A trained team member inserted the CGM sensor into the posterior upper arm at least 24 h before surgery, retaining it for at least 48 h postoperatively. The sensor was removed at least 2 days post-surgery or upon patient discharge, and data were retrieved using Sibionics^®^ Medical software (in.xls format).

### Procedure

Anesthesia induction involved administration of ciprofol (0.3–0.4 mg/kg) as slow bolus injections over approximately 30 s, alongside sufentanil (0.15 μg/kg). Endotracheal intubation was facilitated using rocuronium (0.6 mg/kg). Patients were randomly assigned to receive either an intravenous bolus of 8 mg dexamethasone or a matched placebo (0.9% saline) during anesthesia induction. Anesthesia was maintained with 1.5% sevoflurane in oxygen and a continuous ciprofol infusion (0.8–2.4 mg/kg/h), titrated to sustain a Narcotrend Index (NI) of 37–64 and a mean arterial blood pressure within 20% of baseline. Rocuronium was intermittently administered to maintain neuromuscular blockade. Remifentanil (0.25–4 μg/kg/min) was infused intraoperatively, with optional sufentanil (10–15 μg) at the end of anesthesia at the discretion of the anesthesia team. A standard dose of 4 mg ondansetron was administered at the end of the surgery per institutional practice. Intraoperative management and monitoring adhered to institutional standards, including electrocardiogram (ECG), arterial blood pressure (via radial artery cannulation), pulse oximetry (SpO_2_), and NI (Narcotrend, Monitor Technik, Bad Bramstedt, Germany). All oral antidiabetic agents and insulin were discontinued on the morning of surgery. Intraoperatively, blood glucose was measured every 30–60 min using a standard glucometer (ACCU-CHEK Guide, Roche Diagnostics, Indianapolis, IN), and insulin was administered according to an institutional sliding scale regimen based on the measured glucose values: 0 insulin for glucose < 11.1 mmol/L; 2 units for 11.2–13.9 mmol/L; 3 units for 14.0–16.7 mmol/L; 4 units for 16.8–19.4 mmol/L. Glucose levels were continuously monitored via CGM, while intraoperative clinical decisions were independent of CGM readings. After surgery, patients were transferred to the post-anesthesia care unit, where nausea and vomiting were assessed and ondansetron was administered as rescue antiemetic when needed. Postoperative pain was managed with patient-controlled intravenous opioid analgesia.

### Outcomes

The primary outcome was the maximum intraoperative glucose change, defined as the difference between the highest intraoperative glucose value and the preoperative baseline value (CGM reading immediately before induction of anesthesia), both obtained from CGM.

Key secondary outcomes included perioperative GV metrics [average blood glucose value (Glu Ave), standard deviation (SD), coefficient of variation (CV), and blood glucose instability index (GLI)] assessed intraoperatively and at 12, 24, and 48 h postoperatively. CGM-derived time-in-range metrics (13), [time in range (TIR) 3.9–10.0 mmol/L, time above range (TAR) glucose 10.1–13.9 mmol/L, time above range (TAR) glucose > 13.9 mmol/L, and time below range (TBR) glucose < 3.9 mmol/L] measured on postoperative day (POD)1 and (POD)2. Additional outcomes included CGM accuracy, assessed by comparison with finger-prick capillary blood glucose measurements; quality of recovery on POD1 and POD2 (QoR-15) (14); 48-h incidence of PONV; 48-h incidence of severe pain; pulmonary complications within 2 days postoperatively (Supplementary Table 1) (15); C-reactive protein (CRP) levels on POD1; intraoperative insulin requirements. Patient demographic, clinical, and surgical characteristics were also collected.

Adverse events (AEs) included all device- or dexamethasone-related events. Serious adverse events (SAEs) were defined as death, life-threatening events, significant disability or organ dysfunction, or major events requiring intervention. Clinical trial insurance covering drug-related AEs and SAEs was provided for all participants.

### Sample size

The sample size was calculated using PASS 15.0 software (NCSS, LLC, Kaysville, UT, United States) for a two-sample *t*-test. The primary outcome was the perioperative change in blood glucose levels. Based on a previous study (4), the mean ± SD increase in blood glucose was 3.7 ± 2.7 mmol/L in patients receiving dexamethasone and 1.6 ± 2.1 mmol/L in those not receiving dexamethasone. With a two-sided significance level of 0.05% and 90% power, a minimum of 29 patients per group was required. Accounting for a 10% dropout rate, the calculated sample size was 33 patients per group (66 in total). To further ensure adequate statistical power given the elderly diabetic population, we conservatively expanded enrollment to 36 patients per group, yielding a final total of 72 patients. The formula used by the software was:


$$n=\frac{2\,\sigma^{2}\,{\left(Z_{\alpha /2}+Z_{\beta }\right)}^{2}}{\delta^{2}}$$

where σ is the pooled standard deviation, δ is the expected mean difference, Z_α/2_ = 1.96 for a two-sided 0.05 significance level, and Z_β_ = 1.28 for 90% power.

### Statistical analysis

Perioperative GV was assessed using the following metrics: SD was calculated for all glucose values recorded in all patients in each group; CV (16) was calculated as the SD divided by mean glucose value; and GLI represented the squared difference between consecutive blood glucose levels per unit of actual time between samples (17). GV parameters were computed using the validated Easy GV (18) software v10.^1^

Continuous glucose monitoring sensor accuracy was assessed using the mean absolute relative difference (MARD) (19), defined as the mean of the absolute relative differences between CGM and reference glucose values. The 20/20% consistency was defined as the proportion of glucose readings from CGM within ±20% (glucose > 4.4 mmol/L) or ±1.1 mmol/L (glucose ≤ 4.4 mmol/L). Agreement between CGM and reference measurements was further assessed using Bland-Altman analysis. Bias was calculated as the mean difference between paired values, with 95% limits of agreement defined as bias ±1.96 × SD of the differences.

Efficacy analyses were conducted in the modified intention-to-treat (mITT) population, comprising all randomized patients who underwent surgery and had available outcome data. Continuous variables were expressed as mean ± SD or median (IQR), with group differences assessed using a two-tailed *t*-test or Mann-Whitney U-test. Categorical variables were expressed as numbers and percentages, and intergroup comparisons were made using Pearson’s chi-square test or Fisher’s exact test. To assess the robustness of the primary and key secondary glycemic outcomes, multivariable linear regression was performed for each glycemic endpoint, with dexamethasone as the independent variable and baseline glucose, BMI, HbA1c, insulin use, duration of one-lung ventilation, intraoperative fluid administration and extent of resection as covariates. Each glycemic parameter was analyzed as a dependent variable in separate models. All analyses were performed using SPSS statistical software (version 29.0; IBM Corp., Armonk, NY, United States), with a two-sided *P*-value < 0.05 considered statistically significant.

## Results

### Participants and baseline characteristics

**FIGURE 1:**
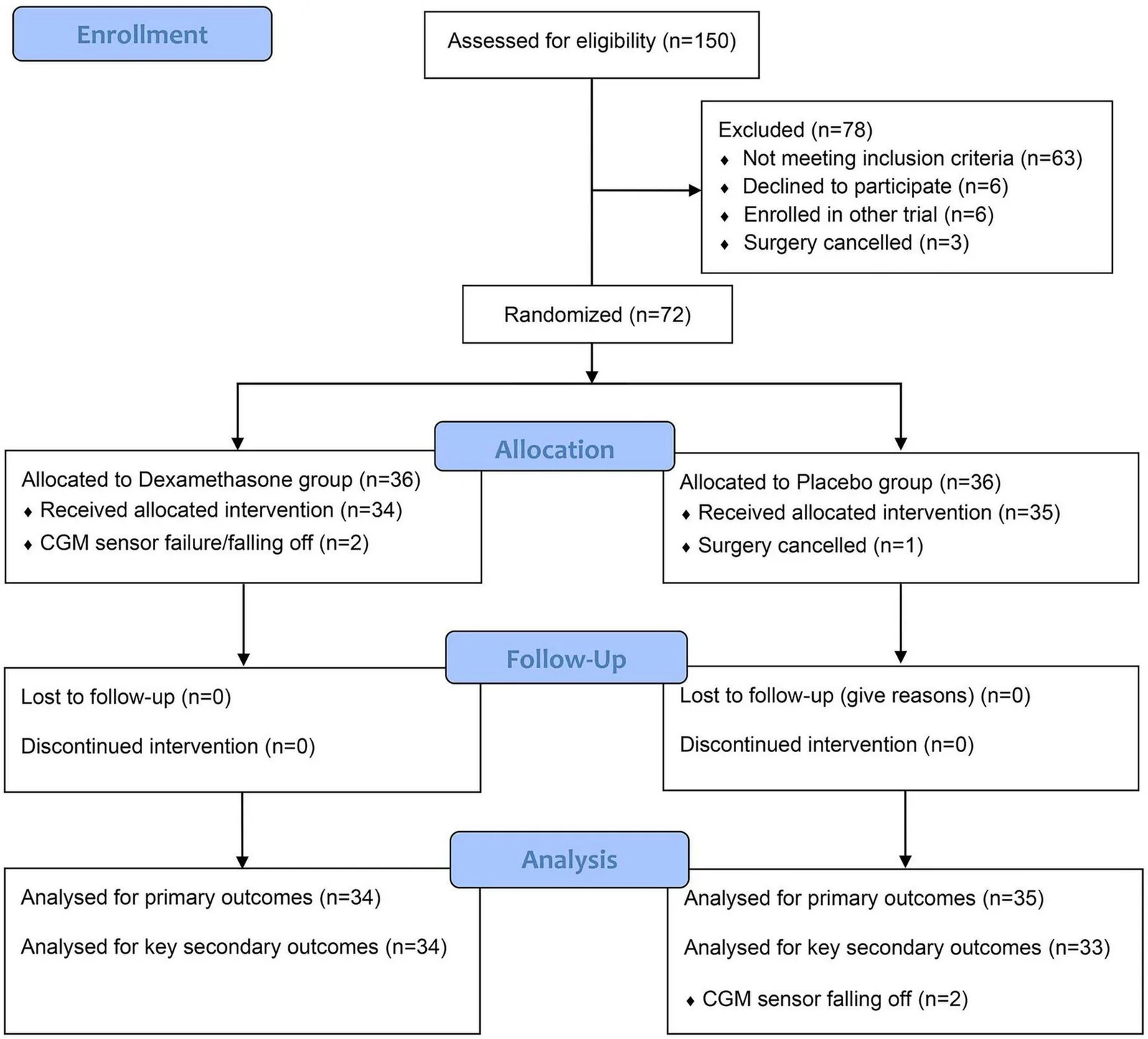
Study flow diagram. CGM, continuous glucose monitoring. Study flow diagram adapted from the CONSORT 2010 statement.

Between November 2023 and May 2024, 150 elderly patients with T2DM were screened (Figure 1); 72 were randomized to either the dexamethasone or placebo group. For primary outcome, two patients in the dexamethasone group experienced CGM technical failures or sensor detachment, while one surgery in the placebo group was canceled due to preoperative hyperglycemia. There were no missing key secondary outcome data for the dexamethasone group. However, two patients in the placebo group were excluded due to CGM detachment. Baseline characteristics and patient data were similar between the two groups. The two groups were well-matched in age, with median ages of approximately 68.5 years in both groups (Table 1).

**TABLE 1:** Personal data and baseline characteristics.

Variable	Dexamethasone group (*n* = 34)	Placebo group (*n* = 35)	*P-*value
Age (years)	68.5 ± 4.9	68.5 ± 5.7	0.973
Female	16 (47.1)	21 (60.0)	0.281
BMI (kg/m^2^)	24.1 ± 2.6	25.1 ± 3.3	0.170
ASA physical status			0.417
2	28 (82.4)	26 (74.3)	–
3	6 (17.6)	9 (25.7)	–
Laboratory test results			
Leukocyte count (10^9^/L)	6.3 (4.9–8.1)	6.5 (5.2–7.4)	0.728
Preoperative glucose (mmol/L)	6.2 ± 1.4	6.0 ± 1.2	0.529
HbA1c (%)	6.8 (6.4–7.4)	6.8 (6.0–7.3)	0.327
Preoperative diabetes treatment^a^			0.088
None	5 (14.7)	2 (5.7)	–
Insulin	13 (38.2)	7 (20.0)	–
Oral	15 (44.1)	21 (60.0)	–
Unknown	1 (2.9)	5 (14.3)	–
SGLT-2 inhibitor	3 (8.8)	7 (20.0)	0.306
Preoperative comorbidities			
Hypertension	19 (55.9)	22 (62.9)	0.555
Coronary heart disease	3 (8.8)	7 (20.0)	0.329
History of stroke	6 (17.6)	4 (11.4)	0.695
Other^b^	7 (20.6)	10 (28.6)	0.442
Diabetes-related complications	3 (8.8)	4 (11.4)	0.517
QoR-15^c^	135 (135–135)	135 (134–137)	0.937
Extent of resection			0.705
Wedge resection	17 (50.0)	14 (40.0)	–
Segmentectomy	4 (11.8)	5 (14.3)	–
Lobectomy	13 (38.2)	16 (45.7)	–
Intraoperative data			
Patients requiring insulin	2 (5.9)	1 (2.9)	0.489
Insulin administered (units)	0.0 (0.0–0.0)	0.0 (0.0–0.0)	0.541
Duration of one-lung ventilation (min)	79.5 (51.0–130.3)	100.0 (71.0–136.0)	0.141
Length of Surgery (min)	120.5 (78.8–180.0)	130.0 (95.0–175.0)	0.449

^a^No patients were documented to be using GLP-1 receptor agonists.

^b^Other preoperative comorbidities mainly included chronic obstructive pulmonary disease (COPD), kidney stones, cholecystolithiasis, benign prostatic hyperplasia, tinnitus.

^c^The tool comprises 15 questions within five dimensions: pain, physical comfort, physical independence, emotional status and psychological support; ASA, American Society of Anesthesiologists; HbA1c, glycated hemoglobin; QoR-15, 15-item quality of recovery.

### Primary and key secondary outcomes

For the primary outcome, maximum intraoperative glucose change was significantly higher in patients receiving dexamethasone than in those receiving placebo (*P* = 0.009; Table 2).

**TABLE 2:** Primary, key secondary outcomes.

Variable	Dexamethasone group (*n* = 34)	Placebo group (*n* = 35)	*P*-value
Primary outcome			
Intraoperative maximal glucose change (mmol/L)	3.6 (3.3–4.9)**	3.1 (2.6–4.0)	0.009
Key secondary outcomes	*n* = 34	*n* = 33	
Intraoperative			
Glu Ave (mmol/L)	5.4 (4.6–6.4)	5.5 (4.8–6.0)	0.960
Glu SD (mmol/L)	1.1 (1.0–1.4)***	0.8 (0.7–1.0)	<0.001
CV (%)	21.9 ± 6.3***	15.8 ± 6.1	<0.001
GLI (mmol^2^/L^2^/h)	3.0 (1.7–4.1)***	1.4 (0.9–2.4)	<0.001
12 h after surgery			
Glu Ave (mmol/L)	9.4 (7.7–10.9)	8.0 (7.4–9.4)	0.069
Glu SD (mmol/L)	1.5 (1.1–1.9)	1.4 (1.0–2.0)	0.861
CV (%)	16.1 (11.7–19.5)	18.4 (13.0–22.9)	0.168
GLI (mmol^2^/L^2^/h)	1.4 (1.1–2.1)	1.5 (1.0–2.9)	0.890
24 h after surgery			
Glu Ave (mmol/L)	9.1 (7.6–11.2)	8.7 (7.6–9.5)	0.256
Glu SD (mmol/L)	2.0 ± 0.7	1.8 ± 0.6	0.214
CV (%)	20.9 (16.7–24.3)	19.5 (15.4–23.2)	0.178
GLI (mmol^2^/L^2^/h)	1.8 (1.2–3.0)	1.9 (1.1–2.5)	0.773
% TAR > 13.9 (mmol/L)	0.0 (0.0–3.7)	0.0 (0.0–2.1)	0.424
% TAR (10–13.9) (mmol/L)	24.1 (10.6–48.2)	18.7 (4.2–27.9)	0.158
% TIR (3.9–10) (mmol/L)	72.5 (46.2–88.3)	77.6 (68.1–95.7)	0.129
% TBR < 3.9 (mmol/L)	0.0 (0.0–0.0)	0.0 (0.0–0.0)	0.775
48 h after surgerya			
Glu Ave (mmol/L)	9.0 (7.9–9.7)	8.2 (7.5–9.3)	0.216
Glu SD (mmol/L)	2.2 (1.7–2.6)	1.7 (1.6–2.5)	0.262
CV (%)	21.8 ± 7.3	20.3 ± 5.8	0.388
GLI (mmol^2^/L^2^/h)	2.0 (1.5–2.8)	2.0 (1.5–2.7)	0.954
% TAR > 13.9 (mmol/L)	0.4 (0.0–5.9)	0.5 (0.0–5.1)	0.678
% TAR (10–13.9) (mmol/L)	24.1 (14.5–45.7)	18.6 (7.8–27.7)	0.095
% TIR (3.9–10) (mmol/L)	70.2 (57.9–83.9)	77.4 (65.2–91.8)	0.142
% TBR < 3.9 (mmol/L)	0.0 (0.0–0.0)	0.0 (0.0–0.0)	0.964

^a^48 h after surgery data available: dexamethasone group (*n* = 33), placebo group (*n* = 33). Glu Ave, average blood glucose value; Glu SD, blood glucose standard deviation; CV, coefficient of variation; GLI, blood glucose instability index; TIR, time in range; TAR, time above range; TBR, time below range.

**P < 0.01, ***P < 0.001 compared to placebo group.

Among the intraoperative GV metrics, Glu Ave values were comparable between groups (*P* = 0.960). In contrast, SD, CV, and GLI were all significantly higher in patients receiving dexamethasone (all *P* < 0.001). GV remained comparable between the two groups at 12, 24, and 48 h postoperatively, as assessed by Glu Ave, SD, CV and GLI (all *P* > 0.05) (Table 2).

Multivariate analysis showed that dexamethasone administration independently predicted greater intraoperative maximum blood glucose change after adjusting for baseline glucose levels, BMI, HbA1c, insulin use, duration of one-lung ventilation, intraoperative fluid administration and extent of resection (Supplementary Table 2). Dexamethasone also independently predicted higher intraoperative GV levels, as reflected by SD, CV, and GLI (all *P* < 0.05; Supplementary Table 2).

During the 24-h postoperative period, the proportion of time spent within the target glucose range (TIR 3.9–10.0 mmol/L) was similar in both groups (*P* = 0.117). There were no occurrences of hyperglycemia above 13.9 mmol/L or hypoglycemia below 3.9 mmol/L. Over 48 h postoperatively, no significant differences were observed between groups in TAR > 13.9, TAR 10.0–13.9, or TIR 3.9–10.0 mmol/L (all *P* values > 0.05). No time was spent in TBR < 3.9 mmol/L (Table 2).

### Perioperative data

Intraoperative and post-anesthesia care unit management parameters were comparable between groups (Supplementary Table 3). Intraoperative insulin requirements were similar between the two groups (Table 1). Among secondary endpoints, CRP levels on POD1 were significantly lower in the dexamethasone group (*P* = 0.015; Table 3). The incidence of PONV was numerically lower with dexamethasone (11.8% vs. 20.0%), but this difference did not reach statistical significance (*P* = 0.350; Table 3). No significant differences were observed in other perioperative clinical outcomes, including pulmonary complications, QoR-15 scores, or severe pain (Table 3 and Supplementary Table 4).

**TABLE 3:** Postoperative data and safety outcomes.

Variable	Dexamethasone group (*n* = 34)	Placebo group (*n* = 35)	*P*-value
Composite pulmonary complications within 2 days	9 (26.5)	11 (31.4)	0.650
Postoperative bleeding	1 (2.9)	2 (5.7)	1.000
Severe pain 0–48 h after surgery^a^	10 (29.4)	11 (31.4)	0.856
PONV 0–48 h after surgery	4 (11.8)	7 (20.0)	0.350
QoR-15 24 h after surgery	109.3 ± 11.0	104.9 ± 11.2	0.102
QoR-15 48 h after surgery	122.6 ± 7.8	120.9 ± 6.8	0.352
Length of postoperative stay (day)	4.0 (3.8–5.0)	4.0 (3.0–5.0)	0.991
Entire length of stay (day)	8.0 (7.0–10.0)	8.0 (7.0–11.0)	0.730
Laboratory test results			
CRP concentration at day 1 (mg/dL)	20.0 (12.3–43.9)*	41.6 (21.1–74.2)	0.015
Leukocyte count day 1 (10^9^/L)	12.9 ± 3.2	11.8 ± 3.1	0.158
CGM sensorb			
CGM sensor fall-off	1 (2.8)	2 (5.7)	1.000
Technical failure of CGM sensor	1 (2.8)	0 (0.0)	1.000
CGM sensor related bleeding	1 (2.8)	0 (0.0)	1.000
Surgical-site infection	0 (0.0)	0 (0.0)	–
Diabetic ketoacidosis	0 (0.0)	0 (0.0)	–
Hyperglycemia hyperosmolar state	0 (0.0)	0 (0.0)	–
Serious adverse events	0 (0.0)	0 (0.0)	–

^a^Severe pain was defined as the subject’s subjective report of an 8, 9, or 10 pain intensity on a vertical, 10-point visual analog scale (VAS) scores.

^b^CGM sensor data available: dexamethasone group (*n* = 36), placebo group (*n* = 36). PONV, postoperative nausea and vomiting; CRP, C-reactive protein; CGM, continuous glucose monitoring.

*P < 0.05 compared to placebo group.

### Accuracy for continuous glucose monitoring

**FIGURE 2:**
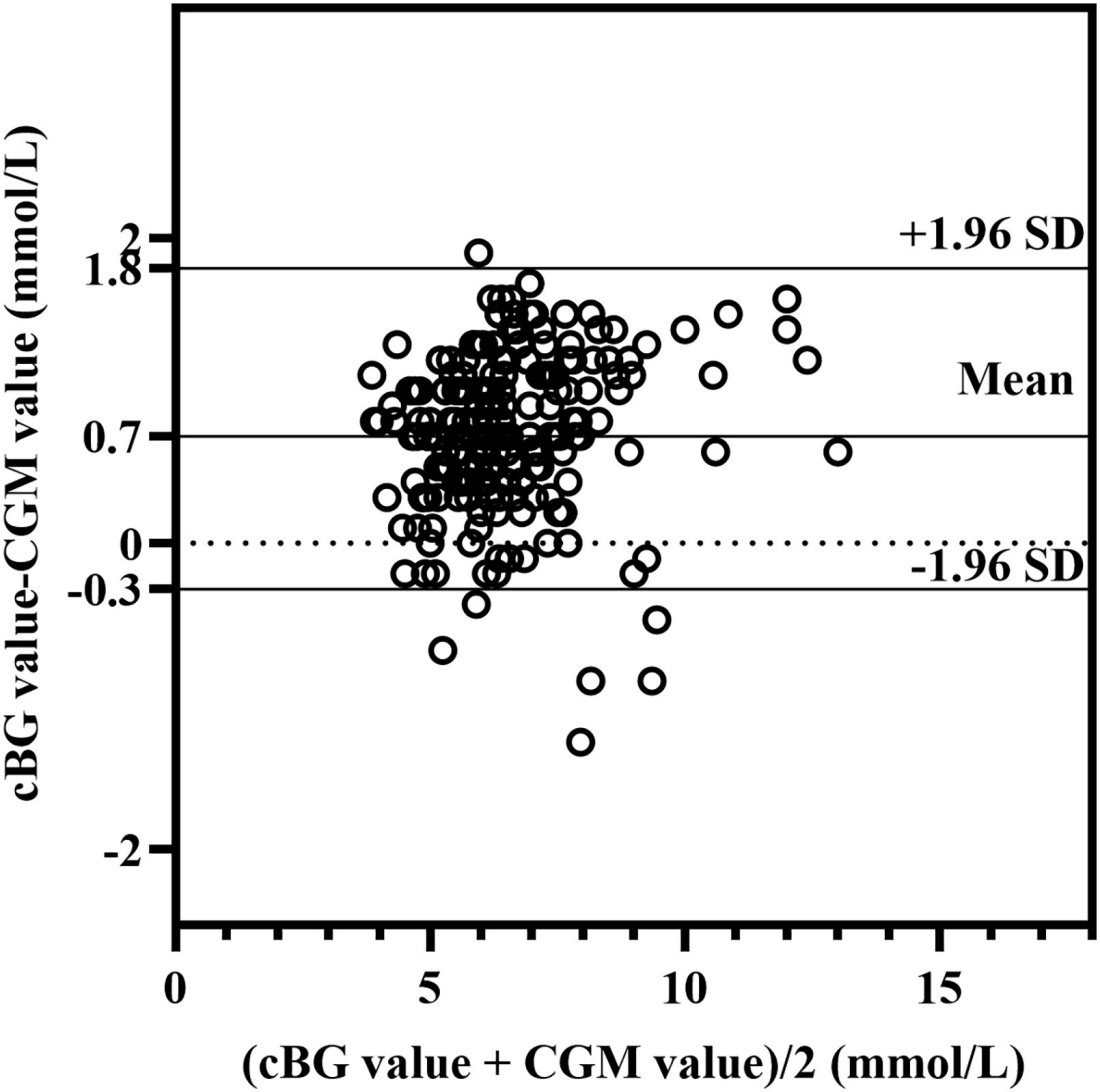
Bland–Altman plot. In Bland–Altman analysis, the mean of paired values is on the X-axis and the difference is on the Y-axis. CGM, continuous glucose monitoring; c BG, finger prick capillary blood glucose; SD, standard definition.

During the intraoperative period, 207 paired glucose values were collected using CGM and finger capillary testing. The 20/20% consistency rate was 88.4% (183/207), and the median overall MARD% was 11.5% ± 5.9%. Bland–Altman analysis revealed a mean bias of 0.7 mmol/L, with 95% limits of agreement ranging from −0.3 to 1.8 mmol/L (Figure 2). Of the 207 paired values, 200 (96.6%) fell within the 95% limits of agreement.

### Safety outcomes

No AEs related to dexamethasone administration or any SAEs occurred in either group. CGM sensor-related events included unexpected detachment (one in the dexamethasone group), technical sensor failure (one in the dexamethasone group), and minor bleeding at the sensor site (one in the dexamethasone group) (Table 3).

## Discussion

In this study, elderly patients with T2DM who underwent elective video-assisted thoracoscopic lung resection and received 8 mg of intravenous dexamethasone experienced greater maximum intraoperative blood glucose change and increased GV compared to those who received a placebo. However, at the other postoperative time points, there were no significant differences in GV or CGM-derived glucose distribution between the groups.

Although it reportedly increases blood glucose levels in patients with diabetes (4, 20, 21), high-quality randomized controlled trials assessing dexamethasone’s effects on blood glucose in elderly individuals are lacking. Nonetheless, meta-analyses have showed that dexamethasone-induced hyperglycemia begins within 2 h of anesthesia induction, with reported peaks within 24 h postoperatively ranging from approximately 0.4–1.6 mmol/L (7, 22). In the present study, patients receiving dexamethasone exhibited a median intraoperative maximum glucose change of 3.6 mmol/L, which was 0.5 mmol/L higher than that observed in the placebo group (3.1 mmol/L). Specifically, while mean blood glucose levels continued to rise until 48 h after surgery, the glycemic effect of dexamethasone was primarily concentrated intraoperatively. In contrast, several studies have reported no significant glycemic effect of dexamethasone in patients with diabetes or those with well-controlled diabetes (8, 23). These discrepancies may reflect differences in patient selection, surgical procedures, or glucose assessment methods—notably, previous research has largely relied on intermittent blood glucose monitoring, which fails to capture the full extent of perioperative glycemic fluctuations.

This study provides the first detailed characterization of dexamethasone-induced glycemic dynamics in older patients with diabetes using CGM. Beyond confirming an increase in intraoperative glucose change, CGM-derived metrics of glycemic variability—SD, CV, and GLI—revealed that dexamethasone also alters the pattern of glucose fluctuations, with a granularity not achievable by intermittent blood glucose monitoring. These findings extend the known glycemic effects of dexamethasone and demonstrate the capacity of CGM to detect clinically meaningful alterations in glucose dynamics in the perioperative setting.

The intraoperative GV observed with dexamethasone likely reflects a transient synergy between the pharmacological effects of the drug and the acute neuroendocrine stress induced by surgery. Surgical procedures elicit a robust counterregulatory hormone response that enhances hepatic glucose output and promotes insulin resistance. Dexamethasone, a synthetic glucocorticoid, exacerbates this already compromised metabolic state by stimulating hepatic gluconeogenesis and impairing peripheral glucose disposal (24). In older patients with T2DM, whose β-cell reserve is already diminished (25), this combined perturbation may temporarily exceed their limited capacity to buffer glucose fluctuations, resulting in the heightened GV observed intraoperatively. Postoperatively, as acute surgical stress diminishes and endogenous counterregulatory hormone levels decline, the sustained pharmacological effect of dexamethasone alone is insufficient to produce detectable differences in GV between groups. It also elucidates why a prior study (8) that relied on intermittent glucose monitoring reported no significant glycemic effect of dexamethasone: without the high temporal resolution of CGM, these transient, stress-dependent fluctuations would remain undetectable.

Perioperative hyperglycemia and increased GV have been associated with adverse outcomes in surgical patients, including infection, major adverse events, and postoperative delirium (26). Recent meta-analyses (27) suggest that perioperative dexamethasone use in patients with diabetes does not increase the risk of postoperative infection. Consistent with these findings, the present trial did not detect significant differences in clinical outcomes such as pulmonary complications. Therefore, the observed increase in intraoperative blood glucose changes and GV should be considered risk markers rather than surrogates for clinical outcomes. Beyond its glycemic effects, dexamethasone significantly reduced C-reactive protein levels on postoperative day 1, consistent with its known anti-inflammatory action (28). Although a trend toward reduced PONV was observed in the dexamethasone group, the difference was not statistically significant, and no significant differences were detected in recovery quality (29) or postoperative analgesia (30). These clinical findings should be interpreted with caution, given that the study was powered for glycemic rather than clinical outcomes.

Since its introduction in 2,000, CGM technology has substantially improved accuracy and reliability, leading to its widespread clinical adoption (31). According to National Medical Products Administration guidelines (32), the target for 20/20% consistency is 65% (clinical standard 60%), and the MARD% target is 18% (clinical standard 20%). In the current study, CGM achieved 20/20% consistency of 88.4% and a MARD% of 11.5%, while slightly lower than previously reported values (91.82% and 8.83 ± 4.03%, respectively) (12), still represent acceptable accuracy. These differences may be attributed to minor variations between fingertip capillary and laboratory blood glucose measurements, even after correction as well as physiological delays of 6–10 min between interstitial and capillary compartments during rapid glucose changes (33). The accuracy may have also been compromised by common perioperative confounders (31).

## Limitations

This study has several limitations. First, only patients with relatively stable preoperative glycemic control were enrolled, which may limit generalizability to those with poorly controlled diabetes. Second, CGM accuracy may be reduced immediately after sensor placement due to local inflammatory responses. To minimize this risk, the sensor was inserted 24 h in advance, as current CGM devices employ improved algorithms to correct the post-insertion variability. Third, the treating anesthesiologist was not blinded to group allocation, which introduces a theoretical risk of performance bias. However, several design features mitigated this risk. Intraoperative insulin administration followed a standardized, protocol-based algorithm, limiting discretionary dosing. More importantly, all clinical decisions were guided by conventional point-of-care capillary glucose measurements, whereas the CGM-derived outcome data were managed solely by the research team and were not accessible to the treating clinician. This separation of the clinical and research data streams prevented any bias in clinical management from directly affecting the study endpoints. Furthermore, any unconscious bias toward more aggressive insulin therapy in the dexamethasone group would have attenuated rather than exaggerated the observed glycemic differences, making our significant findings conservative estimates. Fourth, the study was powered only for glycemic outcomes. Therefore, the lack of significant differences in clinical outcomes—including infectious complications—should be interpreted with caution, and whether the observed increase in glycemic metrics translates into clinical harm warrants investigation in larger, adequately powered studies. Finally, this trial did not evaluate different dexamethasone doses or include individuals without diabetes. Hence, future CGM-based research should examine dose-dependent effects and compare outcomes in elderly individuals with and without diabetes.

## Conclusion

In conclusion, in older patients with type 2 diabetes undergoing thoracoscopic lung resection, a single 8 mg dose of dexamethasone increased intraoperative maximum glucose change and GV, without significantly affecting overall perioperative time in range. CGM captured these transient perturbations in detail and demonstrated acceptable accuracy, thereby revealing a dimension of dexamethasone-induced glycemic instability not detectable by conventional intermittent monitoring. Whether these fluctuations translate into detectable clinical consequences in those with poor glycemic control warrants investigation in larger trials with clinical endpoints.

## Data Availability

The original contributions presented in this study are included in this article/Supplementary material, further inquiries can be directed to the corresponding author.

## Footnotes

1
https://www.phc.ox.ac.uk/research/resources/easygv
