# Supplementary material for: Single-dose dexamethasone increases perioperative glycemic variability in older patients with diabetes undergoing lung surgery: a continuous glucose monitoring-based randomized controlled trial
Source: Front Med (Lausanne). 2026 Aug 27;13:1880028. doi: 10.3389/fmed.2026.1880028 (PMC13558395; doi:10.3389/fmed.2026.1880028)
Supplement: Supplementary file 1 [file Table_1.pdf]

Supplementary Material

Supplementary Table 1 Definition of major pulmonary complications.

	**Definition**
Pneumonia	Defined as the presence of a new or progressive radiographic infiltrate plus at least two of three clinical features including fever of > 38°C, leukocytosis or leukopenia, and purulent secretions
Pneumothorax	Defined as air in the pleural space with no vascular bed surrounding the visceral pleura
Pleural effusion	Defined as chest radiograph demonstrating blunting of the costophrenic angle, loss of sharp silhouette of the ipsilateral hemidiaphragm in upright position, evidence of displacement of adjacent anatomical structures or (in supine position) a hazy opacity in one hemithorax with preserved vascular shadows
Atelectasis	Defined as lung opacification with a shift of the mediastinum, hilum or hemidiaphragm toward the affected area, and compensatory over-inflation in the adjacent non atelectatic lung
Respiratory failure	Defined as a postoperative PaO_2 <60 mmHg on room air, a PaO_2/FiO_2 ratio < 300 mmHg or arterial oxyhemoglobin saturation measured with pulse oximetry <90% and requiring oxygen therapy
Requirement for mechanical ventilation	Defined as unplanned need of non–invasive or invasive ventilation

Supplementary Table 2 Multivariable linear regression of factors associated with intraoperative glycemic profiles.

	Coefficient (95% CI)	Standard Error of the Coefficients	*P* value
**Maximum blood change**			
Dexamethasone use	0.941 (0.290-1.592)	0.326	0.005
Baseline glucose levels	-0.008 (-0.274-0.259)	0.133	0.954
BMI levels	0.014 (-0.094-0.121)	0.054	0.799
HbA1c (%)	0.454 (0.013-0.895)	0.220	0.044
Insulin use	-0.276 (-0.990-0.428)	0.357	0.386
Duration of one-lung ventilation	0.002 (-0.005-0.008)	0.003	0.629
Fluid administration	0.001 (-0.000-0.003)	0.001	0.166
Extent of resection	0.227 (-0.133-0.586)	0.180	0.212
R^2	0.254		
Adjusted R^2	0.155		
F-statistic	2.554		0.018
**SD**			
Dexamethasone use	0.386 (0.196-0.575)	0.095	< 0.001
Baseline glucose levels	-0.015 (-0.093-0.062)	0.039	0.692
BMI levels	0.001 (-0.030-0.033)	0.016	0.925
HbA1c (%)	0.136 (0.008-0.265)	0.064	0.038
Insulin use	-0.081 (-0.289-0.128)	0.104	0.442
Duration of one-lung ventilation	0.000 (-0.002-0.002)	0.001	0.991
Fluid administration	0.000 (-0.000-0.001)	0.000	0.193
Extent of resection	0.059 (-0.045-0.164)	0.052	0.261
R^2	0.308		
Adjusted R^2	0.216		
F-statistic	3.343		0.003
**CV**			
Dexamethasone use	6.398 (3.183-9.614)	1.608	< 0.001
Baseline glucose levels	0.045 (-1.271-1.360)	0.657	0.946
BMI levels	-0.292 (-0.824-0.240)	0.266	0.277
HbA1c (%)	0.229 (-1.948-2.405)	1.088	0.834
Insulin use	-2.026 (-5.550-1.498)	1.762	0.255
Duration of one-lung ventilation	-0.002 (-0.035-0.030)	0.016	0.899
Fluid administration	0.005 (-0.003-0.012)	0.004	0.230
Extent of resection	-0.422 (-2.195-1.351)	0.886	0.636
R^2	0.254		
Adjusted R^2	0.154		
F-statistic	2.547		0.019
**GLI**			
Dexamethasone use	1.386 (0.388-2.385)	0.499	0.007
Baseline glucose levels	-0.092 (-0.500-0.317)	0.204	0.655
BMI levels	0.019 (-0.146-0.185)	0.083	0.814

HbA1c (%)	0.564 (-0.112-1.240)	0.338	0.100
Insulin use	-0.515 (-1.609-0.579)	0.547	0.350
Duration of one-lung ventilation	-0.011 (-0.022- -0.001)	0.005	0.026
Fluid administration	0.001 (-0.001-0.004)	0.001	0.253
Extent of resection	0.141 (-0.410-0.692)	0.275	0.610
R^2	0.230		
Adjusted R^2	0.128		
F-statistic	2.247		0.036

Note: HbA1c, glycated hemoglobin; SD, blood glucose standard deviation; CV, coefficient of variation; GLI, blood glucose instability index.

Supplementary Table 3 Perioperative data

Variable	Dexamethasone Group (n=34)	Placebo Group (n=35)	*P* value
Intraoperative data			
Ciprofol (mg)	140.0 (113.8-174.3)	150.0 (108.0-220.0)	0.244
Sufentanil (μg)	30.0 (30.0-35.0)	35.0 (25.0-35.0)	0.604
Remifentanil (mg)	0.9 (0.6-1.5)	1.0 (0.7-1.6)	0.468
Rocuronium (mg)	90.0 (70.0-105.0)	100.0 (80.0-110.0)	0.164
Fluid administration (ml)	1000 (500-1000)	1000 (500-1000)	0.131
Blood loss (ml)	10.0 (8.8-20.0)	10.0 (10.0-20.0)	0.456
Interventions for hemodynamic events	14 (41.2)	16 (45.7)	0.704
Length of anesthesia (min)	153.5 (118.8-216.3)	166.0 (127.0-220.0)	0.490
PACU data			
Medications to treat hypertension	1 (2.9)	4 (11.4)	0.356
Medications to treat hypotension	1 (2.9)	0 (0.0)	0.493
Medications to treat PONV	0 (0.0)	1 (2.9)	1.000
Pain medication	0 (0.0)	1 (2.9)	1.000
Time in PACU (min)	60.5 (36.3-100.0)	64.0 (44.0-102.0)	0.501

Note: PONV, postoperative nausea and vomiting; PACU, post anesthesia care unit.

Supplementary Table 4 Components of pulmonary complications.

Variable	Dexamethasone Group (n=34)	Placebo Group (n=35)	P value
Components of pulmonary complications			
Pneumonia	7 (20.6)	6 (17.1)	0.714
Pneumothorax	0 (0.0)	2 (5.7)	0.493
Pleural effusion	1 (2.9)	3 (8.6)	0.614
Atelectasis	1 (2.9)	1 (5.7)	1.000
Respiratory failure	0 (0.0)	1 (2.9)	1.000
Requirement for mechanical ventilation	1 (2.9)	1 (2.9)	1.000
